# A drug repurposing screen identifies hepatitis C antivirals as inhibitors of the SARS-CoV2 main protease

**DOI:** 10.1371/journal.pone.0245962

**Published:** 2021-02-01

**Authors:** Jeremy D. Baker, Rikki L. Uhrich, Gerald C. Kraemer, Jason E. Love, Brian C. Kraemer

**Affiliations:** 1 Division of Gerontology and Geriatric Medicine, Department of Medicine, University of Washington, Seattle, WA, United States of America; 2 Geriatrics Research Education and Clinical Center, Veterans Affairs Puget Sound Health Care System, Seattle, WA, United States of America; 3 Thomas Jefferson High School, Auburn, WA, United States of America; 4 Western Washington Pathology, Tacoma, WA, United States of America; 5 Department of Psychiatry and Behavioral Sciences, University of Washington, Seattle, WA, United States of America; 6 Department of Pathology, University of Washington, Seattle, WA, United States of America; Cornell University, UNITED STATES

## Abstract

Effective SARS-CoV-2 antiviral drugs are desperately needed. The SARS-CoV-2 main protease (Mpro) appears as an attractive target for drug development. We show that the existing pharmacopeia contains many drugs with potential for therapeutic repurposing as selective and potent inhibitors of SARS-CoV-2 Mpro. We screened a collection of ~6,070 drugs with a previous history of use in humans for compounds that inhibit the activity of Mpro *in vitro* and found ~50 compounds with activity against Mpro. Subsequent dose validation studies demonstrated 8 dose responsive hits with an IC50 ≤ 50 μM. Hits from our screen are enriched with hepatitis C NS3/4A protease targeting drugs including boceprevir, ciluprevir. narlaprevir, and telaprevir. This work suggests previous large-scale commercial drug development initiatives targeting hepatitis C NS3/4A viral protease should be revisited because some previous lead compounds may be more potent against SARS-CoV-2 Mpro than boceprevir and suitable for rapid repurposing.

## Introduction

SARS-CoV-1 and SARS-CoV-2, the cause of the COVID-19 pandemic, are zoonotic coronaviruses found in bats that can infect humans. Initial symptoms of SARS-CoV-2 infection include fever, myalgia, cough, and headache. Infection usually resolves without active medical intervention, but for a subset of cases infection can progress to viral pneumonia and a variety of complications including acute lung damage leading to death [1]. While complications are atypical in most cases, mortality rates increase dramatically with the age and impaired health of infected patients. To date, much of our knowledge of COVID-19 virology has been inferred from the study of similar Severe Acute Respiratory Syndrome (SARS) coronavirus (SARS-CoV-1) and related coronaviruses including Middle East Respiratory Syndrome (MERS-CoV) [reviewed in 2]. Like all coronaviruses, SARS-CoV-2 exhibits an enveloped ribonucleoprotein helical capsid containing a single positive-stranded genomic RNA. Infection starts with receptor-mediated virus internalization, uncoating, and translation of the viral genome [3]. Polyprotein cleavage by viral proteases yields a complement of viral structural and accessory proteins. This polyprotein cleavage is mediated by the coordinated activity of two viral proteases. The main viral protease (Mpro), targeted in this drug screen, and the papain-like protease (PLpro) are responsible for endoproteolytic cleavages of viral polyproteins producing functional viral replicase complex [4]. Recent structural biology work has solved the crystal structure of SARS-CoV-2 Mpro yielding structural insights into Mpro function [5, 6].

Antiviral drugs effective for COVID-19 would have a broad impact on global healthcare in the current coronavirus pandemic. Existing antiviral drugs on the market target a wide variety of both RNA and DNA viruses. There is precedence for targeting the protease, as this approach has been successful in treating both HIV-1 and hepatitis C. [reviewed in 7 and 8]. Other conceptual COVID-19 antiviral targets include the host ACE2 receptor, viral replicase, and viral genome encapsidation. However, previous work with other RNA viruses suggest that Mpro function is essential for viral replication and readily targetable using existing technology. Thus, while there are many potentially targetable activities for COVID-19, the coronavirus Mpro seems a likely choice for rapid drug development.

To accelerate drug development we employed a drug repurposing strategy, an approach of utilizing previously approved drugs for new indications [9, 10]. Previous work suggests libraries enriched with known bioactive drug-like compounds provide the best opportunity for finding new lead compounds [11, 12]. Here we performed a drug-repurposing campaign as a rapid and cost effective means to identify candidate hits. We selected a repurposing library composed of structurally diverse and well-characterized and approved drug molecules. The bioavailability, toxicity and efficacy in human therapy has already been demonstrated for these compounds. To screen as much of the available approved drug space as possible in an easily accessible format we chose to screen the Broad Institute Drug Repurposing Library (6070 compounds, see S1 Table) [13]. This represents about half of the approximately 14,000 approved or experimental drugs known to human clinical medicine [14]. There are significant cost and time advantages realized by drug repurposing as it can accelerate the preclinical phase of development and streamline clinical trials to focus on efficacy rather than safety.

Repositioning existing approved drugs with the capacity to inhibit COVID-19 virus replication and infection would be of profound utility and immediately impact health care in the current pandemic. There are no drugs in clinical use specifically targeting coronavirus replication. The major advantage of the approach taken here is that by screening drugs with a history of previous clinical use, we will be focusing on compounds with known properties in terms of pharmacokinetics (PK), pharmacodynamics (PD) and toxicity. Thus, the Broad Repurposing Library we screened consists of compounds suitable for rapid translation to human efficacy trials.

## Results

### Development of fluorescent Mpro assays

**Fig 1:**
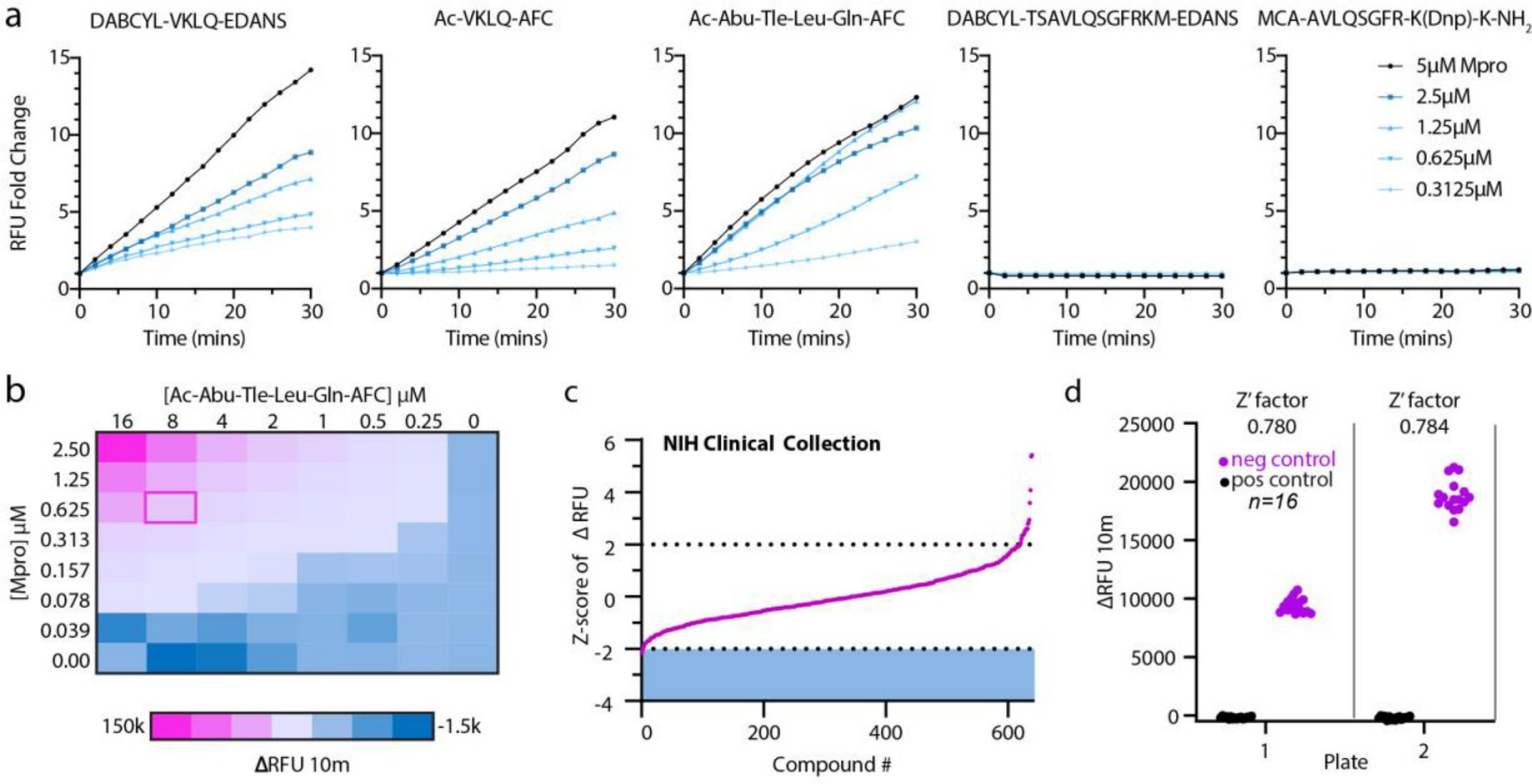
Mpro assay optimization. (a) Screen of selection of reported Mpro substrates: DABCYL-VKLQ-EDANS, Ac-VKLQ-AFC, Ac-Abu-Tle-Leu-Gln-AFC, DABCYL-TSAVLQSGFRKM-EDANS, MCA-AVLQSGFR-K(Dnp)-K-NH_2_. Fold change of increase in RFU was measured over 30 minutes holding substrate constant at 12.5μM with increasing concentrations of Mpro recombinant protein as indicated. (b) Two-dimensional titration of substrate Ac-Abu-Tle-Leu-Gln-AFC against Mpro. Concentrations are indicated for substrate along the x-axis and for Mpro along the y-axis. Heat map corresponds to the change in RFU over 10 minutes and pink outline (ΔRFU at 10 minutes = 20,810 relative fluorescence units) indicates chosen concentration for NIH Clinical Collection screen (0.625μM Mpro and 8μM Substrate). (c) Z-score index of NIH Clinical Collection screen (640 compounds). Hit window was considered at Z-score ≤ -2 and was calculated as the Z-score of ΔRFU at 10 minutes corresponding to the linear portion of the curve. X-axis indicates arbitrary compound number arranged by increasing Z-score. (d) Z’-factor for the two NIH Clinical Collection 384-well plates. Pink circles indicate negative control (DMSO) and black circles represent positive controls (no protein). Z’-factor calculated at 0.780 and 0.784 for plates 1 and 22 respectively. Y axis represents change in RFU over 10 minutes.

We began assay development by selecting potentially suitable synthetic Mpro substrates and compared catalyzed hydrolysis curves between 5 fluorescently labeled substrates (Ac-Abu-Tle-Leu-Gln-AFC [15], DABCYL-VKLQ-EDANS, Ac-VKLQ-AFC, DABCYL-TSAVLQSGFRKM-EDANS [16], and MCA-AVLQSGFR-K(Dnp)-K-NH2) [17]. We chose to use the recently published Ac-Abu-Tle-Leu-Gln-AFC (Abu = 2-Aminobutyrate, Tle = tButylglycine) synthetic non-canonical amino-acid containing peptide as Mpro more readily cleaves this preferred sequence as compared to the native VKLQ sequence [15] (Fig 1A). Substrates DABCYL-TSAVLQSGFRKM-EDANS and MCA-AVLQSGFR-K(DnP)-K-NH2 had drastically lower rates of Mpro catalyzed hydrolysis and were not considered further in our assay development (Fig 1A). To determine concentration ratios between Mpro and substrate, we next preformed a two-dimensional titration and chose 625nM Mpro and 8μM substrate for a balance of relatively modest Mpro protein requirement and a robust fluorescence intensity (Fig 1B). Before screening the Broad library, we piloted our assay conditions against the NIH Clinical collections library (~650 compounds) and calculated our Z’-factor for each plate at 0.780 and 0.784 (Fig 1C and 1D). Z’-factor is a score of suitability of assays for high-throughput screening and is derived from the equation $Z^{’}‐\mathrm{factor}=1-\frac{3(\sigma_{p}+\sigma_{n})}{\left|\mu_{p}-\mu_{n}\right|}$, where σ = standard deviation, μ = mean, p = positive controls, and n = negative controls. A score greater than 0.5 indicates a screenable assay. Although no promising compounds were identified from this smaller library, it demonstrated that our assay was sufficiently robust for screening the much larger Broad Repurposing library.

### Drug repurposing strategy–screening the Broad Repurposing Library

**Fig 2:**
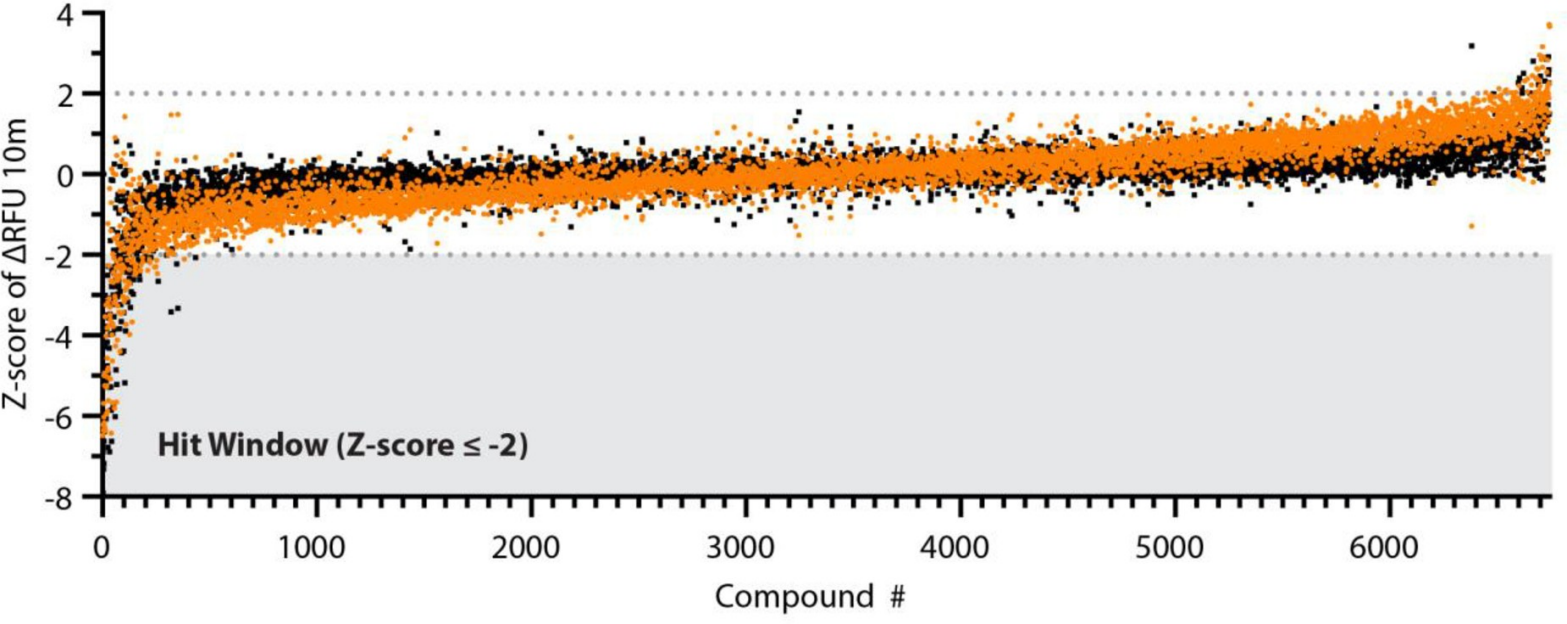
Broad library screen. Screen of the Broad Repurposing Library. Library was screened at a concentration of 50 μM against both Ac-VKLQ-AFC (black) and Ac-Abu-Tle-Leu-Gln-AFC (orange). Hit window was considered for compounds falling below Z-score ≤ -2 against both substrates and consisted of 50 compounds. Compounds ordered by average Z-score.

We acquired this ~6,070 compound library as an assay ready collection in 384-well format. We conducted the library screen at 384-well density using the optimized kinetic Mpro assay described in Fig 1. We conducted a single point screen at 50 μM compound concentration and observed ~50 compounds with activity against SARS-CoV-2 Mpro for an overall hit rate <0.75%. These compounds were screened in parallel against the natural amino acid substrate (Ac-VKLQ-AFC) as well as a kinetically preferred substrate (Ac-Abu-Tle-Leu-Gln-AFC) (Fig 2). Individual compounds are shown in Table 1.

**Table 1:** Hits identified from Broad Repurposing Library screen against SARS-CoV-2 Mpro.

*Common name*	Mechanism of Action	Target	Status
*Mitoquinone*			
*Octenidine*	membrane integrity inhibitor		Launched*
*Boceprevir*	HCV inhibitor	CMA1, CTSA, CTSF, CTSK, CTSL, CTSS	Launched
*STS*		PROC, PROS1	Launched
*NH125*	EEF2 inhibitor		Preclinical
*Visomitin*			
*Cetrimonium*			Launched
*Hexachlorophene*	potassium channel activator	GLUD1, SDHD	Launched
*Benzethonium*	sodium channel blocker	SCN10A	Launched
*Rose-bengal*	contrast agent		Launched
*LGD-6972*			
*Narlaprevir*	HCV inhibitor		Phase 3
*Obatoclax*	BCL inhibitor	BCL2	Phase 3
*Domiphen*			Preclinical
*PSB-06126*	NTPDase inhibitor	ENTPD3	Preclinical
*NSC-95397*	CDC inhibitor	CDC25A, CDC25B	Preclinical
*Deltarasin*	phosphodiesterase inhibitor	KRAS	Preclinical
*Calpeptin*	calpain inhibitor		Preclinical
*TNP-470*	methionine aminopeptidase inhibitor		Phase 2
*TC-LPA5-4*	lysophosphatidic acid receptor antagonist	LPAR5	Preclinical
*Hemin*	enzyme inducer		Launched
*Sennoside*			Preclinical
*PYR-41*	ubiquitin activating enzyme inhibitor		Preclinical
*Telaprevir*	HCV inhibitor	CTSA, PGR	Launched
*Evans-blue*	glutamate receptor modulator	GRIA1, PTPN1	Launched
*C646*	histone acetyltransferase inhibitor	EP300	Preclinical
*NSC-663284*	CDC inhibitor	CDC25A, CDC25B, CDC25C	Preclinical
*TCID*			Preclinical
*Hematoporphyrin*			Launched
*16-BAC*	cationic surfactant		Preclinical
*BMS-833923*	smoothened receptor antagonist	SMO	Phase 2
*AVN-492*			
*Ascorbyl palmitate*			Preclinical
*Elacestrant*			
*Eifuroxazide*			
*Aurothioglucose*	PKC inhibitor	PRKCI	Launched
*Nifursol*	bacterial DNA inhibitor		Launched
*NS-1643*	voltage-gated K^+^ channel activator	KCNH2, KCNH6, KCNMA1	Preclinical
*Tiplaxtinin*	plasminogen activator inhibitor	SERPINE1	Phase 1
*Thiomersal*	antibiotic	OXCT1	Launched
*Indocyanine-green*	contrast agent	SLCO1B1	Launched
*Chlortetracycline*	protein synthesis inhibitor		Launched
*Carbazochrome*			Launched
*Altrenogest*	progestogen hormone	PGR	Launched
*Emricasan*			
*GSK2801*	bromodomain inhibitor	BAZ2A, BAZ2B	Preclinical
*Anthralin*	DNA synthesis inhibitor		Launched
*Melphalan*	DNA alkylating agent, DNA inhibitor		Launched
*RITA*	MDM inhibitor	MDM2	Preclinical
*Azeliragon*	RAGE receptor antagonist	AGER	Phase 3

*Launched = compound approved for humans, though may only be approved for veterinary use in some countries

### Analysis of potency

**Fig 3:**
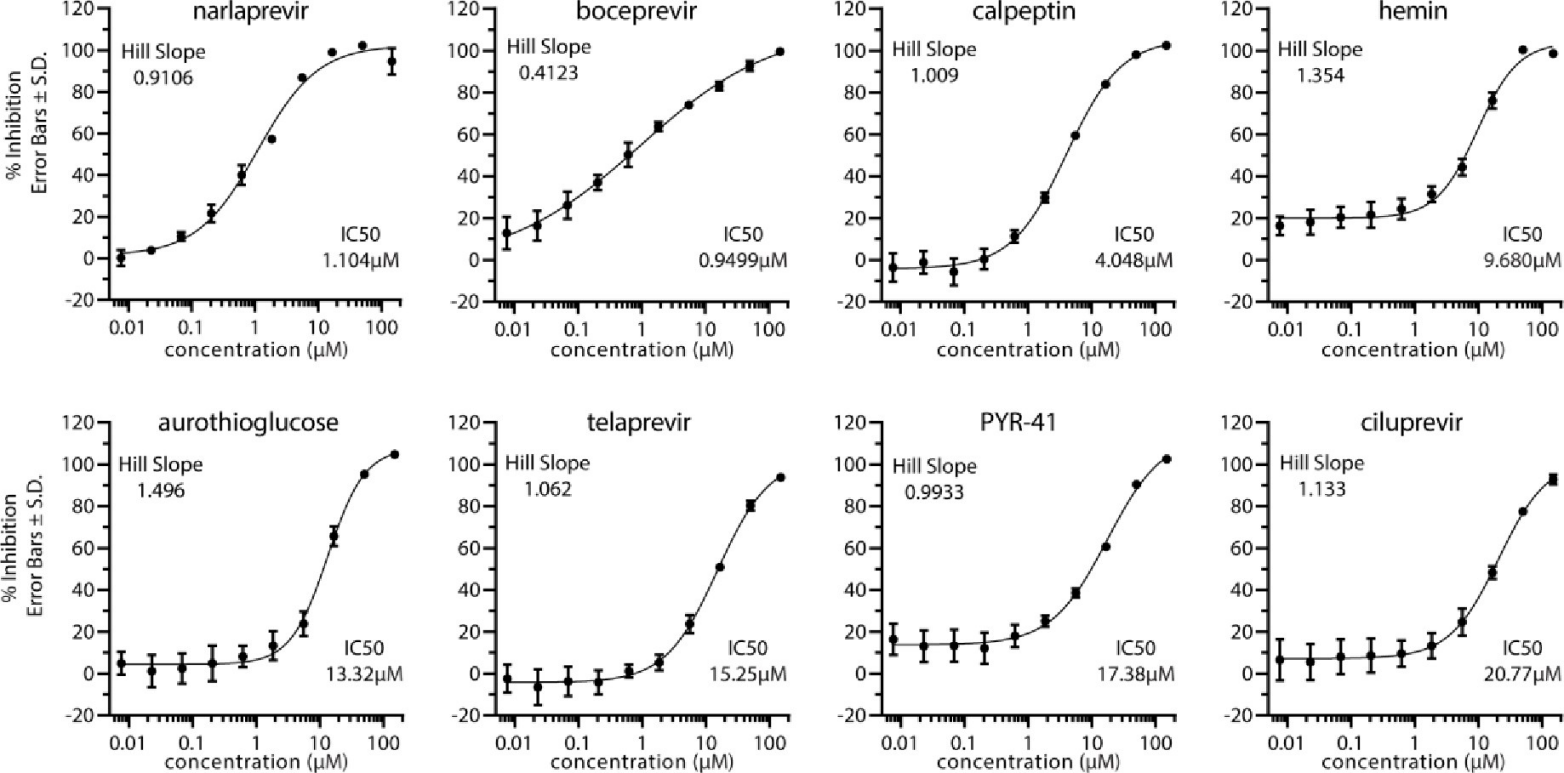
Dose-response validation of compounds against SARS-CoV-2 Mpro. Dose response curves were generated using Ac-Abu-Tle-Leu-Gln-AFC substrate. Percent inhibition of each compound was calculated at indicated 10 concentrations by comparing slope of treatment versus DMSO control. Error bars represent standard deviation and n = 3 for each concentration. IC50 values and Hill Slopes are indicated and calculated by 4-parameter nonlinear regression curve fitting.

We validated the hits from the primary screen by conducting a 10-point dose-response analysis with a drug concentration range from 150 μM down to 7.6 nM (3-fold dilution series). From this dose-response analysis, IC50 values were calculated for dose-responsive hits. Several of the drugs uncovered in our screen including, boceprevir (IC50 = 0.95 μM), ciluprevir (IC50 = 20.77 μM), narlaprevir (IC50 = 1.10 μM), telaprevir (IC50 = 15.25 μM), are antiviral compounds targeting the hepatitis C NS3 protease. boceprevir, narlaprevir, and telaprevir are approved drugs with a track record of safe use in human patients [18–23]. Other relatively potent dose responsive compounds emerging from our screen include calpeptin (IC50 = 4.05 μM), aurothioglucose (IC50 = 13.32 μM), PYR-41 (IC50 = 17.38 μM), and hemin (IC50 = 9.68 μM) (Fig 3). A previous report of ebselen as an inhibitor of Mpro activity could not be replicated in our assay conditions [6]. Ebselen failed to show a significant dose response against Mpro cleavage of our chosen substrate Ac-Abu-Tle-Leu-Gln-AFC (S1 Fig). This may indicate non-specific inhibition of Mpro by ebeselen in some Mpro assay conditions.

### The relative utility of *in silico* and HTS repurposing screens

**Fig 4:**
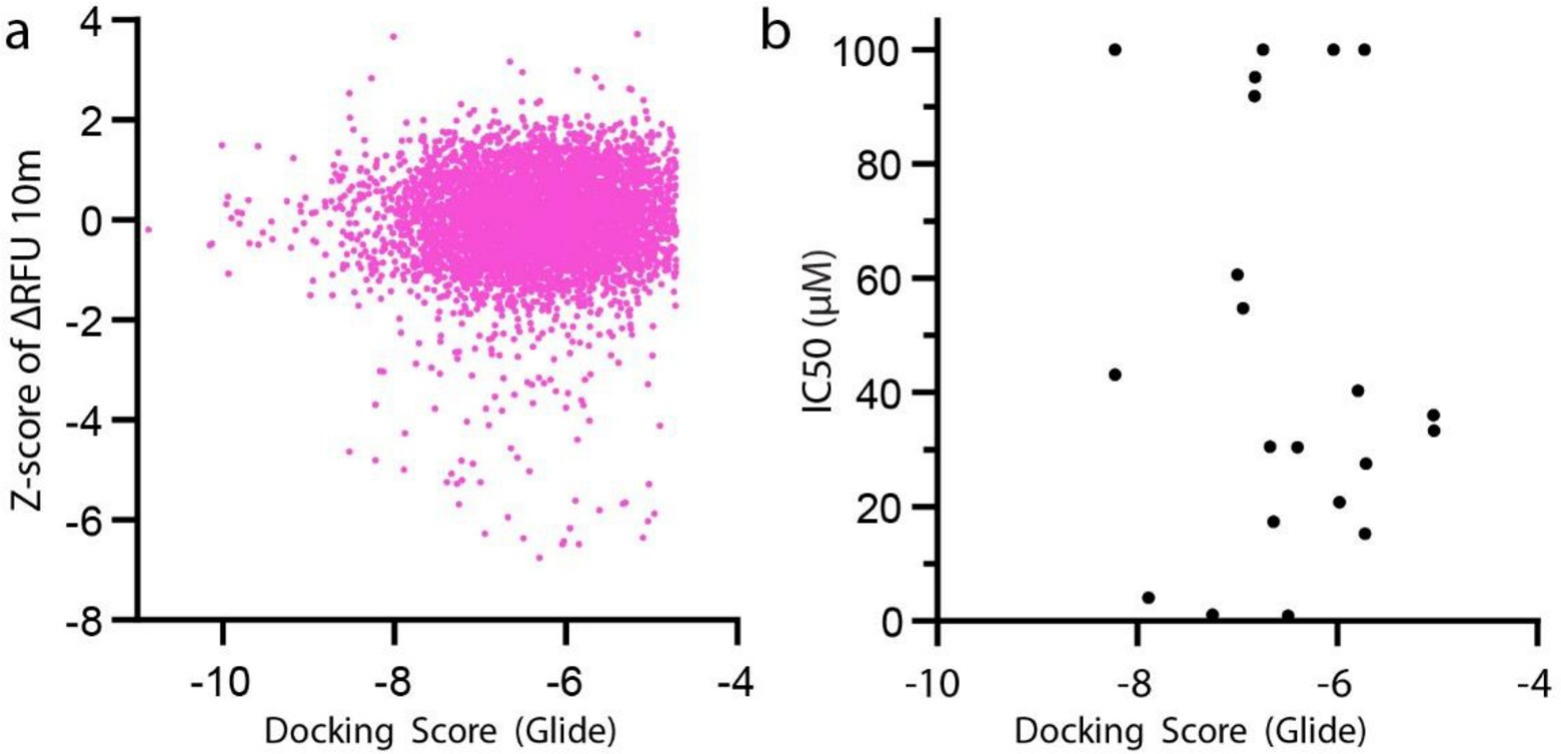
Correlation of experimental and in silico identification. (a) Correlation of Z-score for all Broad Repurposing library compounds and Docking Score. Pearson r = 0.02864 (95% CI 0.003478–0.05376), P value = 0.0257 and XY pairs = 6,068. Linear equation Y = 0.03452x+ 0.2202 as fitted by simple linear regression, slope significantly deviated from 0, P value = 0.0016. (b) Correlation of IC50 vs Docking Score. Pearson r = -0.1053 (95% CI -0.5136–0.3420), P value = 0.6498 and XY Pairs = 21. Linear equation Y = -4.188x+20.41 as fitted by simple linear regression, slope not significantly deviated from 0, P value = 0.6498.

The recent publication of the crystal structure for Mpro has enabled computational approaches to Mpro drug discovery [5, 6]. We leveraged the existing structural data (PBD entry 6LU7) to conduct a computational free energy calculation based *in silico* screening approach. To do this we have utilized the Schrodinger Maestro software package [24, 25] to conduct a computational docking of all compounds in the Broad Repurposing library. Using this approach, we derived a docking score for each compound (see S1 Table for broad repurposing library with docking scores). We observe a poor correlation (Pearson r = 0.02864) between Mpro docking score and Z-score in the protease inhibition assay (Fig 4A). Furthermore, top hits from the screen also exhibit a weak correlation (Pearson r = -0.1503) between compound potency and docking score (Fig 4B).

## Discussion

A general discussion of drug repurposing for COVID-19 suggests viral encoded proteases may be relevant therapeutic targets for coronaviruses [26]. Relative to most other human viruses, our understanding of the virology of SARS-CoV-2 remains incomplete. However, after decades of extensive research, we have learned a great deal about viral proteases in general and the chemical means to inhibit them from our studies of HIV-1, hepatitis C and rhinoviruses. Likewise protease inhibitors targeting the SARS-CoV-1 protease have been investigated [27, 28]. Furthermore, previous work on SARS-CoV-1 Mpro has demonstrated that it is a targetable enzyme worth significant translational effort [29].

Sequence alignment shows a high degree of homology between SARS-CoV-1 Mpro and SARS-CoV-2 Mpro with ~95% amino acid sequence identity. Recent studies have solved the crystal structure of SARS-CoV-2 Mpro and compared it with SARS-CoV-1 Mpro showing that they have similar but distinct active site pockets and will require distinct dugs for potent and highly specific inhibition. This sequence and structural information has provided an opportunity to conduct *in silico* docking of known drugs to the COVID-19 virus Mpro active site. However thus far, such analysis has not uncovered potent inhibitors of Mpro. Recent *in silico* work has suggested that protease inhibitor drugs may inhibit SARS-CoV-2 Mpro [30, 31]. However, our findings suggest that *in silico* approaches alone cannot substitute for enzyme kinetic screening evaluation of Mpro inhibitors because most identified high scoring compounds in *in silico* docking studies lack activity against Mpro in kinetic protease assays.

The objective of this work is to complete a survey of approved drugs to identify therapies that can block COVID-19 viral replication by inhibiting the main viral protease. The advantage of this approach is that any approved drug identified can be advanced rapidly to clinical trials without extensive multi-year preclinical development efforts. To date, only remdesivir has been shown as a potent antiviral medication. The Adaptive Covid-19 Treatment Trial (ACTT-1) was a phase 3 clinical trial which compared remdesivir to placebo in a cohort of 1062 patients. This trial showed a shortened period of recovery for remdesivir treated patients (10 days) versus placebo control (15 days) [32]. The FDA approved remdesivir as the first COVID-19 therapeutic in October 2020. Identification of other potent antiviral treatments targeting different aspects of viral replication will be important for the development of effective combination therapies.

A diverse variety of initial hits were identified in our high throughput screen of the Broad library. Of these, the most potent hits are all known protease inhibitors and there is strong representation from protease inhibitors developed to inhibit HCV protease NS3/4A (boceprevir, ciluprevir, narlaprevir, and telaprevir). Clearly as approved or well-developed clinical candidates, these drugs exhibit pharmacological and pharmacodynamic properties well suited to repurposing as a COVID-19 antiviral therapy. Boceprevir and narlaprevir appear the most potent against Mpro and may be suitable for repurposing. Previous clinical evaluation of boceprevir (also known as Victrelis) showed it to be safe and effective for treating HCV [33]. Boceprevir was approved as a first in class HCV NS3/4A serine protease inhibitor for treatment of chronic HCV infection. Boceprevir was FDA approved for use in the USA in 2011 and boceprevir treatment is given as a combination therapy with interferon α2b and ribavirin. Likewise, clinical evaluation of narlaprevir (also known as Arlansa or SCH900518) showed it to be both safe and to exhibit antiviral activity when combined with interferon α2b [34]. Furthermore, narlaprevir has been show effective against HCV NS3/4A mutations causing resistance to protease inhibitors [35]. Narlaprevir was approved for use against HCV in Russia in 2016.

Our findings demonstrate Boceprevir and Narlaprevir potency against SARS-CoV-2 Mpro in the one micromolar range. Recent work from other labs has also identified boceprevir as an inhibitor of SARS-CoV-2 Mpro enzymatic activity [36, 37]. Further, boceprevir SARS-CoV-2 antiviral activity has been reported at an EC50 of 1.95±1.62 μM [37] in a cellular model of viral replication. It should be noted that the potency for SARS-CoV-2 for Mpro enzymatic inhibition (~1 μM) is reduced compared to the HCV protease, IC50 reported below 90 nM [38, 39]. This 10-fold reduction in potency against enzymatic activity may affect dosages required for potent antiviral activity and underscores the need for examining previous HCV Mpro inhibition campaigns for compounds that may more potently inhibit SARS-CoV-2 Mpro. Further translational studies are required to precisely determine the IC50 for antiviral activity of SARS-CoV-2. Likewise, previous commercially developed NS3/4A inhibitor lead compounds may be suitable for further repurposing studies.

The COVID-19 pandemic has revealed an urgent unmet medical need for potent antiviral agents for treatment of SARS-CoV-2 infection. Because antiviral therapies are frequently most effective when used in combination [40], it may be useful to consider combining Mpro inhibition with other antiviral strategies for treating SARS-CoV-2 infection. For instance, inhibition of the SARS-CoV-2 replicase in combination with Mpro inhibition might exhibit synergistic antiviral activity. Remdesivir, a broad spectrum antiviral replicase inhibitor has shown efficacy against a wide variety of RNA viral replicases including SARS-CoV-2 [41, 42] and was recently FDA approved for COVID-19 after efficacy was shown in a cohort of over 1000 patients. Thus, combination therapies using boceprevir/remdesivir or narlaprevir/remdesivir may yield a synergistic drug repurposing strategy for treating COVID-19. Combination therapy is particularly important given the low genetic barrier of resistance for some HCV inhibitors. For example, point mutations in NS3 protease variants can cause drastic reductions in potency of telaprevir, where EC50 against WT NS3 is reduced up to 60 fold depending on the mutation [38], while a similar study for boceprevir shows single amino acid mutations may cause a reduction in activity of up to 120-fold [43]. Although concerns remain about the potency of current HCV antivirals against SARS-CoV-2 Mpro, work presented here supports the evaluation of compounds identified in previous HCV NS3/4A targeting campaigns for potential repurposing as a COVID-19 therapy.

## Methods

### Recombinant protein

Recombinant Mpro was purified using constructs and methods as previously described [5]. pGEX-6P-1 plasmid containing SARS-CoV-2 Mpro was gifted from Hilgenfeld lab at Luebeck University, Germany. Plasmid was transformed into BL21 (DE3) bacteria (NEB). A single colony was inoculated into 10mL Terrific Broth (TB) + Carbenicillin (25μg/mL) and grown overnight to saturation. Overnight culture was transferred into 1L of TB and grown in a shaking incubator at 37°C until log phase (OD_600_~0.7). Culture was induced with IPTG (1mM final) and kept in 37°C shaking incubator for 4 hours. Culture was spun down at 3,400rpm for 30 min at 4°C, and pellet resuspended in PBS with 10% sucrose then spun at previous conditions. PBS was aspirated and bacteria pellet was snap frozen in liquid nitrogen and stored at -70°C. Pellet was thawed and resuspended in Lysis buffer (PBS, 0.3% lysozyme, 1mM DTT, 1.5% Sarkosyl, RNAse A, and DNAse I) and sonicated for 10 seconds ON time, 20 seconds OFF time for 5 minutes of total ON time at 60% amplitude. Lysate was spun at 16,000rpm for 30 minutes at 4°C. 4mL of Ni-NTA beads and supernatant were rotated for 2 hours at room temperature. Gravity column was used for purification with His_6_-tagged Mpro binding to Ni-NTA beads (Qiagen 30210), washed with lysis buffer + 10mM imidazole and eluted with increasing concentration of imidazole (50mM, 100mM, 150mM and 200mM). The majority of Mpro eluted at between 150-200mM imidazole and was 90%+ pure by Coomassie stained gel analysis. Mpro fractions were pooled and buffer exchanged into 20mM Tris pH 7.8, 150mM NaCl, 1mM EDTA, 1mM DTT, and snap frozen in liquid nitrogen and stored at -70°C. Yield and purity were assessed via BCA (ThermoFisher 23225) and Coomassie-stained SDS-PAGE.

### Fluorescent Mpro protease assay

Fluorescent Mpro peptides were synthesized by Anaspec (www.anaspec.com) at 90% purity and frozen in 1 mg aliquots. Stock concentration of substrates were made by reconstituting powder in 100μL DMSO (10mg/ml) and storing at -70°C. Optimal Mpro substrates were previously determined to be Ac-Val-Lys-Leu-Gln-AFC for physiological substrates and Ac-Abu-Tle-Leu-Gln-AFC, a noncanonical amino acid substrate [15]. Ac-Val-Lys-Leu-Gln-AFC and Ac-Abu-Tle-Leu-Gln-AFC fluorogenic substrates were monitored at 380/20 nm excitation and 500/20 nm emission wavelengths. FRET-based substrates Dabcyl-Val-Lys-Leu-Gln-EDANS was measured at 336/20 nm excitation and 490/20 nm emission wavelengths and MCA-Ala-Val-Lys-Gln-Ser-Gly-Phe-Lys-DNP-Lys was monitored at 325/20 nm excitation and 392/20 nm emission wavelengths. We used 20mM Tris pH 7.8, 150mM NaCl, 1mM EDTA, 1mM DTT, 0.05% Triton X-100 as the assay buffer. Assay conditions were at room temperature (25°C) for all assays.

### 2D titration main screen optimization

2D titration for determining the main screen ratios was done in 96 well black opaque plates (Corning 3686 NBS). The top concentration of Mpro was 2.5μM and serial diluted to 0.0395μM along the Y-axis of the plate. The top concentration of substrate was 16μM and serial diluted along the X-axis of the plate. Fluorescence was monitored at 380/20 nm excitation and 500/20 nm emission wavelengths.

### Broad Repurposing Library

The Broad Repurposing Library was ordered and plated into black opaque 384-well plates (Greiner 781209) at 100nL of 10mM (slight variations depending on compound) compound in DMSO. 10μL of diluted Mpro (625nM final concentration in reaction buffer detailed above) was added with a MultiFlo FX liquid dispenser using a 5μL cassette. Compounds were incubated with Mpro for 10 minutes at RT after which 10uL of substrate (8μM final concentration of either Ac-VKLQ-AFC or Ac-Abu-Tle-Leu-Gln-AFC) was dispensed into the plate and read using a Cytation 5 multi-mode reader immediately at 380/20 nm excitation and 500/20 nm emission wavelengths every 5 minutes for 30 minutes. Data was analyzed using Biotek Gen5 software, Microsoft Excel, and GraphPad Prism 8.

### Dose validation assays

Hit compounds were ordered from the Broad Institute pre-plated in 384-well format (Greiner 781209) as 10-point serial dilutions (3-fold) at 300nL per well. Mpro (80nM final concentration) and substrate (Ac-Abu-Tle-Leu-Gln-AFC at 32μM final concentration) were dispensed in the same manner described above. Inhibition was calculated as $1-\frac{\Delta RFU(10m)sample}{\Delta RFU(10m)control}$ at each concentration and data fitted to 4-parameter nonlinear regression model using GraphPad Prism 8.

### *In silico* docking of the Broad Repurposing Library with Mpro

We utilized the Schrodinger Maestro software package [24, 25] to conduct a computational docking of all compounds in the Broad Repurposing library. In this approach we generated a receptor grid model of the Mpro active site and serially docked each compound in the Broad Repurposing library with the active site model using the physics based Glide algorithm [44, 45]. We chose the Glide algorithm over the many competing options because of its superior performance in head to head comparisons of algorithms [44].

### Statistical analyses, IC50 calculation, HTS suitability score (Z’-factor), and correlation

Graphs were generated using GraphPad Prism 8. For the primary screen, 2 individual data points for each compound are shown, one point for each of two different substrates Ac-VKLQ-AFC or Ac-Abu-Tle-Leu-Gln-AFC. Inhibition for each compound was calculated as $1-\frac{\Delta RFU(10m)sample}{\Delta RFU(10m)control}$. Z-score for compound inhibition was calculated as $Z=\frac{x-\mu }{\sigma }$, where *x* = individual sample value, *μ* = sample mean, and *σ* = sample standard deviation. Hits were considered at Z-score ≤ -2. X-axis indicates arbitrary compound number arranged by increasing Z-score. For dose response validation, curves consist of data points for 10 concentrations of each compound run in triplicate (n = 3) and inhibition was calculated as stated above. Error bars represent standard deviation. IC50 values and Hill slope values were calculated using GraphPad by fitting to 4-parameter nonlinear regression. The statistic, Z’-factor was used to evaluate suitability of high-throughput screening (HTS) design [46] and is defined by equation $Z^{’}‐\mathrm{factor}=1-\frac{3(\sigma_{p}+\sigma_{n})}{\left|\mu_{p}-\mu_{n}\right|}$, where *σ* = standard deviation, *μ* = mean, *p* = positive controls, and *n* = negative controls. An ideal assay exhibits a Z’-factor value of 1, while a value of 1 > Z ≥ 0.5 indicates a large separation band between positive and negative controls, showing the assay is robust and suitable for screening and identification of potential inhibitors. When the Z’-factor value is below 0.5, assays are less reliable exhibiting decreased control separation and increased signal variation. Correlation analysis was conducted using GraphPad by fitting data to simple linear regression and calculating Pearson Coefficients as shown.

## Supporting information

S1 Fig Dose response curves for ebselen against Mpro cleavage of substrate Ac-Abu-Tle-Leu-Gln-AFC. (TIF)

S1 Table Broad Library SDF with docking scores. (XLSX)
