# Supplementary figures and images for: A drug repurposing screen identifies hepatitis C antivirals as inhibitors of the SARS-CoV2 main protease
Source: PLoS One. 2021 Feb 1;16(2):e0245962. doi: 10.1371/journal.pone.0245962 (PMC7850479; doi:10.1371/journal.pone.0245962)

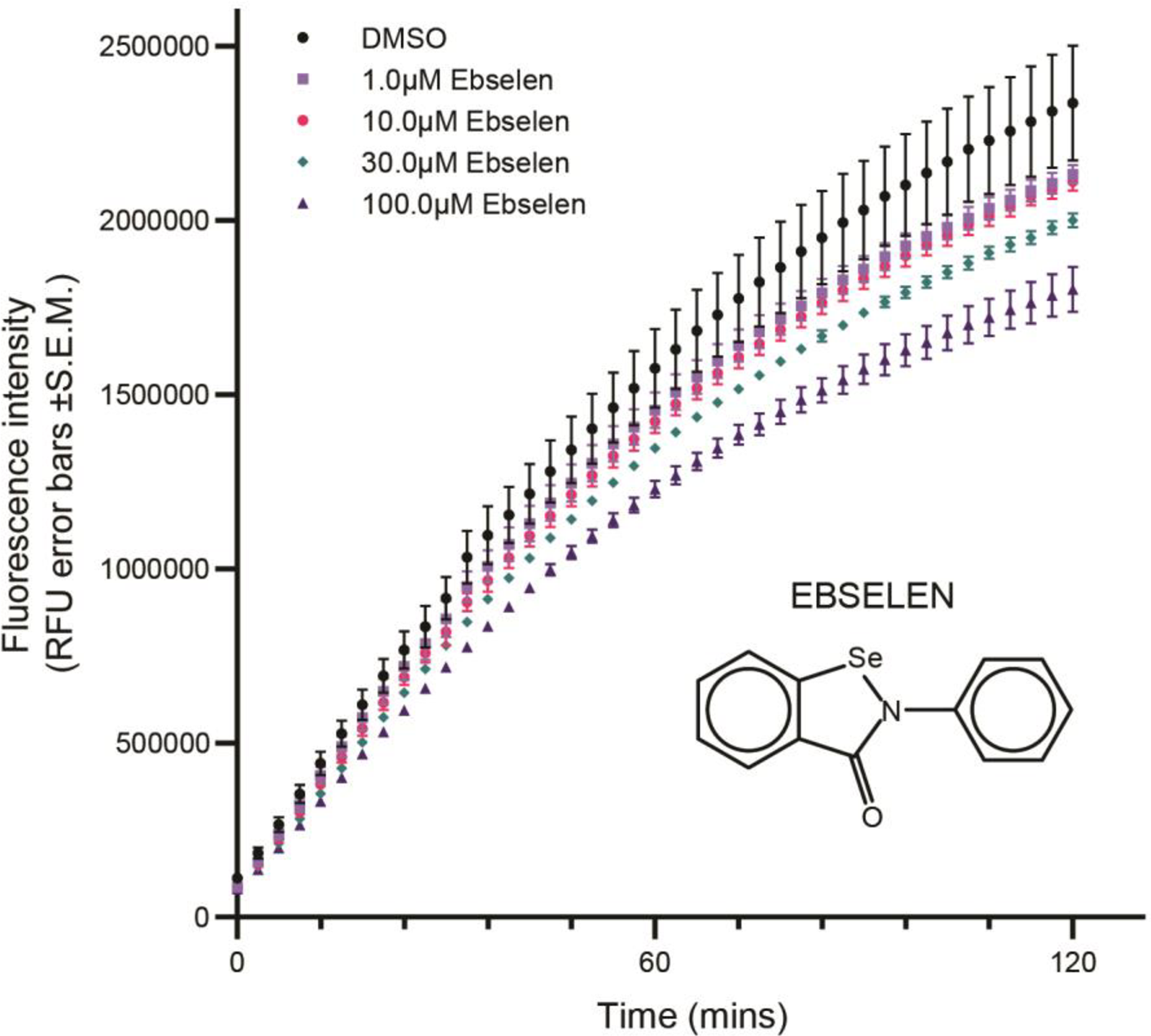

Supplement: S1 Fig — (TIF) [file pone.0245962.s001.tif]
